# Why Use Low-Carat Solder on New Dental Work?

**Published:** 1905-05-15

**Authors:** William H. Trueman

**Affiliations:** Philadelphia


### WHY USE LOW-CARAT SOLDER ON NEW DENTAL WORK?

BY WILLIAM H. TRUEMAN, D.D.S., PHILADELPHIA.

The time was, and not so very long ago, when gold solder kept for sale at the dental depots was so brittle and so broken that it might be handled with a scoop. This is now changed. Firms catering to the wants of dental mechanics have brought skill and science to bear upon their metallurgical departments, and as a result the needed precious metals are obtainable in better form and in greater variety. The tendency however, to debase the solders, while no doubt in response to a demand for solder of easy flowing qualities, is not to be commended. The fault, however, is not with them, but is due to faulty teaching in our schools and text-books. The time was when solders of a standard carat were for sale; now they are advertised and branded, not of as a certain carat, but as *solder for a certain carat plate*, special stress being laid upon their easy-flowing qualities and good color rather than upon their fineness. The reduced price plainly indicates that they are debased.

In an excellent text-book upon prosthetic dentistry, now before me, I find the following formula for a gold solder, which, it states, "may be used in connection with eighteen or twenty carat plate:"

6 dwts. pure gold.
2 dwts. rosette copper.
1 dwt. fine silver.

That such a formula should be published in a dental text-book of so recent a date (1897) is a disgrace, and yet it is a fair sample of those set before dental students by their class teachers and their text-books in this year of grace, 1905, copied without change from ancient text-books.

In the first place, it is not explicit, nor yet in accord with usage in scientific works. Why should the reader be compelled to work out a mathematical problem in order to obtain information that should be conveyed by the formula itself?

Let it read:

16 parts (or dwts. or grain, it is immaterial which) pure gold,

$5\frac{1}{3}$ parts rosette copper,

$2\frac{2}{3}$ parts fine silver, and the reader knows at once that the resulting solder is sixteen carats fine. It may be used, of course, upon eighteen or twenty carat plate; but why should such low carat solder ever be used in connection with eighteen or twenty carat plate? This is followed by two others, one a fraction over fifteen carats fine, and another eighteen, both naming gold coin and brass as components. Now let me ask, What is "rosette copper?" What is "gold coin?" What is "brass?" When this formula was first written, some seventy or more years ago, "rosette copper" might possibly have been some special brand of that metal; it has no meaning now. Brass, fine brass, brass pins, etc., are of as uncertain composition as is a low grade of boarding-house hash. Regarding "gold coin," nothing is certain beyond its containing a definite amount of pure gold. No doubt, when the mouth blow-pipe was in general use, with the well-remembered alcohol lamp, that now and again blew up, or more frequently "went out," like those of the unwise virgins, and from the same cause, before the soldering operation was completed, or in prolonged operations blinded one by its irritating fumes, a solder fusing at a few degrees less heat was appreciated. All that is now of the past, and it is high time that expedients made necessary by meager facilities were erased from dental college teaching and dental text-books.

All the needed metals, gold, silver, copper and zinc, are now readily attainable, as nearly absolutely pure as chemical processes carefully conducted can make them; and they only should be used. The difference in cost between pure gold and pure silver, and coin gold and coin silver, is trifling. Pure gold costs, at the refiners, one dollar and ten cents per dwt. for any amount less than one ounce, and twenty-one dollars an ounce. This never changes. Pure silver fluctuates; its present price is about seventy-five cents an ounce; pure copper is about twenty cents an ounce; pure zinc about fifteen cents an ounce. The base metals may be obtained from dealers in fine chemicals, who provided these metals for use in delicate chemical processes. There is no excuse for using, and no excuse for advising the use of commercial metals or alloys in making either plate or solder for dental use. They all contain harmful impurities.

Now, it is not every dentist who has facilities for making his own plate or solder, and when it can be purchased from reliable parties it is better to do so. It is well, however, to understand that eighteen carat gold solder and solder for eighteen carat gold plate are not the same. The latter is usually about two points lower carat.

If in the formulas for plate and solder given in the text-books the number of grains of the precious metal in each pennyweight is first given, the formula itself announces the carat. It is rather more scientific to state the parts in thousands; either, however, is explicit. The use of alloys should be discarded. There is no advantage in their use; they are confusing and unreliable.

The following formulas I use in my own laboratory. They are all I need for new work, and I find them quite satisfactory. They flow freely, have good color, and are as tough and pliable as plate. Between their fusing point and that of the metals for which they are intended there is ample margin. The eighteen carat will flow freely upon silver plate, and the twenty-two carat upon eighteen carat plate without in either case the plate being over-heated. This is my test of their fusibility.

Twenty-two carat gold solder:

22 parts pure gold.

1 part pure copper.

$1\frac{1}{4}$ parts pure zinc.

Eighteen carat gold solder:

18 parts pure gold.
3 parts pure silver.
2 parts pure copper.
$1\frac{1}{2}$ parts pure zinc.

Silver solder:

19 parts pure silver.
1 part pure copper.
4 parts pure zinc.

There is a slight loss of zinc, especially in making gold solder, not from oxidation, as the books generally state, but from volatilization, which is quite a different thing.

A fraction of a grain is added to allow for this. If pure metals are used, there is so little oxidation that as a rule the crucible from which the solder is poured is left perfectly clean; so clean, indeed, that they are promptly broken to avoid the temptation of using them again; a dangerous experiment, as the borax usually penetrates the bottom and causes them to leak when used a second time.

Either in safety to the porcelain teeth or to the plate, the slight difference in the fusing point between the high and low grade solder is of no practical advantage, and for new work a solder of a lower carat than the plate is inexcusable. The lower grade solders are occasionally required in making repairs and should be used for that purpose only.

Silver being more fusible requires a solder a little less fine, but there is no excuse for using a silver solder composed of equal parts of silver plate and brass.

While zinc is a base metal, and is credited with causing brittleness when alloyed with the precious metals the character it has acquired as a component of precious metal alloys is not deserved. It is due to the impurities found associated with it rather than to the metal itself.

Notwithstanding that the solder sold as solder for eighteen carat plate is but little if any finer than sixteen carat, I am told by dealers that many dentists when purchasing eighteen carat gold plates purchase sixteen carat solder to use with it, so that as a matter of fact they are using on new work fourteen carat solder. I wonder why! It cannot be economy; the difference in cost is too insignificant. I take it to be ignorance, or it may be that they do not possess proper heating arrangements, or lack effective blowpipes.

With the advent of crown and bridge-work, a handy form of blow pipe has come into general use. For small, delicate operations they are very convenient, especially when the work is done in the office on an office work-bench. None that I have tested have proved satisfactory for serious work. The old-fashioned large gas flame and the old-fashioned blow pipe, operated with a foot pump or bellows, may make things hot on a warm day, may occasionaly singe the hair or burn the fingers, but when their use is once mastered, soldering with honest, high-grade solder is a pleasure.

As gold solders are now graded, a solder two carats higher than the plate should always be used. It would be better, however, if the dealers would cease to equivocate and sell their gold solders for what they really are.

Another reform. Our text-book writers should discard all the old out-of-date formulas. There is no necessity for a multiplicity of formulas, one for each grade is amply sufficient; and they should teach that the plate and solder should be of the same fineness.

As before remarked, the difference in the fusing point of solder differing slightly in carat is no help to the workman constructing a new denture, but it makes a great deal of trouble at times, when that piece of work requires repairs or alterations, and these are attempted with "honest" solder.—*Office and Laboratory.*

